# Analysis of the Gas Phase Acidity of Substituted Benzoic Acids Using Density Functional Concepts

**DOI:** 10.3390/molecules25071631

**Published:** 2020-04-02

**Authors:** Jorge A. Amador-Balderas, Michael-Adán Martínez-Sánchez, Ramsés E. Ramírez, Francisco Méndez, Francisco J. Meléndez

**Affiliations:** 1 Departamento de Fisicomatemáticas, Facultad de Ciencias Químicas, Av. San Claudio y 14 Sur, Col. San Manuel, Benemérita Universidad Autónoma de Puebla, C.P. 72570, Puebla, Pue., Mexico; 2 Departamento de Química, División de Ciencias Básicas e Ingeniería, Universidad Autónoma Metropolitana-Iztapalapa, A.P. 55-534, Mexico, D.F., 09340 Mexico; 3 Le Studium Guest Research Fellow, Loire Valley Institute for Advanced Studies, 4500 Orleans & Tours, France; 4 Conditions Extrêmes et Matériaux: Haute Température et Irradiation (CEMHTI), UPR3079 CNRS, CEMHTI 1, Avenue de la Recherche Scientifique, 45071 Orleans, France; 5 Departamento de Fisicoquímica, Facultad de Ciencias Químicas, Av. San Claudio y 14 Sur, Col. San Manuel, Benemérita Universidad Autónoma de Puebla, C.P. 72570, Puebla, Pue., Mexico

**Keywords:** absolute gas phase acidity, Fukui function, chemical potential, softness, nucleophile, electrophile, inductive effect, resonance effect, polarizability effect

## Abstract

A theoretical study of the effect of the substituent Z on the gas phase acidity of substituted benzoic acids ZC_6_H_4_COOH in terms of density functional theory descriptors (chemical potential, softness and Fukui function) is presented. The calculated gas phase Δ_acid_*G*° values obtained were close to the experimental ones reported in the literature. The good relationship between the Δ_acid_*G*° values and the electronegativity of ZC_6_H_4_COOH and its fragments, suggested a better importance of the inductive than polarizability contributions. The balance of inductive and resonance contributions of the substituent in the acidity of substituted benzoic acids showed that the highest inductive and resonance effects were for the -SO_2_CF_3_ and -NH_2_ substituents in the *para*- and *ortho*-position, respectively. The Fukui function confirmed that the electron-releasing substituent attached to the phenyl ring of benzoic acid decreased the acidity in the trend *ortho* > *meta* > *para***,** and the electron-withdrawing substituent increased the acidity in the trend *ortho* < *meta* < *para*.

## 1. Introduction

The Hammett equation (log*K*_a_^X^/log*K*_a_^H^ = *σρ*) is a linear Gibbs free energy relationship (a link between equilibrium constant and reaction rates) that uses the properties of substituted benzoic acids as a reference standard to study the effect of substituent on the reactivity and properties of various molecules [1]. Although the Hammett equation is fulfilled in the *meta*- and *para*-substituted benzoic acids (the correlation of acidity with the *σ* scale is obtained), it is not obeyed for the *ortho*-isomers [2,3]. Exner et al. [3] have suggested that the main part of *ortho* effects in the acidity of substituted benzoic acids is due to inductive and resonance effects and steric effects are of limited importance for acidity. Verevkin et al. [4] evaluated the thermochemical properties of methylbenzoic and methoxybenzoic acids and found that the conformational peculiarities of the *ortho*-methoxybenzoic acid have not relevance for the enthalpy of formation of this molecule. Taft et al. [5,6,7], Exner et al. [8,9] and Geerlings et al. [10,11] have pointed out the usefulness of the polarizability to the analysis of structural effects in proton-transfer reactions in gas phase. Chattaraj et al. [12] found trends of changes in acidity of *meta*- and *para*-substituted benzoic acids with electrophilicity based charge transfer index, fractional number of electrons transferred and group charge. Liu et al. [13] employed molecular electrostatic potential and the valence natural atomic orbitals to predict the acidity of 196 singly, doubly, and triply substituted benzoic acids. (the *ortho*-benzoic acids were excluded). Vianello and Maksic [14] found that the acidity in a series of *para*-substituted benzoic acids increased with the stability of the corresponding conjugate bases. Hollingsworth et al. [15] examined the effects of substituents on the p*K*as of a set of 16 *meta*- and *para*-substituted benzoic acids using density functional theory [B3LYP/6-311G(d, p)] calculations, they found a good correlations with the Löwdin, Mulliken, AIM, and natural population analysis charges on atoms of the dissociating carboxylic acid group. Méndez et al. studied the effect of substituent on the acidity and reactivity of *para*-substituted phenols [16,17,18], ethanol derivatives [19] and their anions and found that the extension of the charge dispersal in the anions was the key to understanding the gas-phase acidity [17,20]. In addition to the analytical equations, developed from the density functional theory (DFT) [21] and the hard and soft acids and bases (HSAB) principle [22,23] showed the mathematical relationship between the gas phase acidity and the hydrogen charge [16].

**Figure 1:**
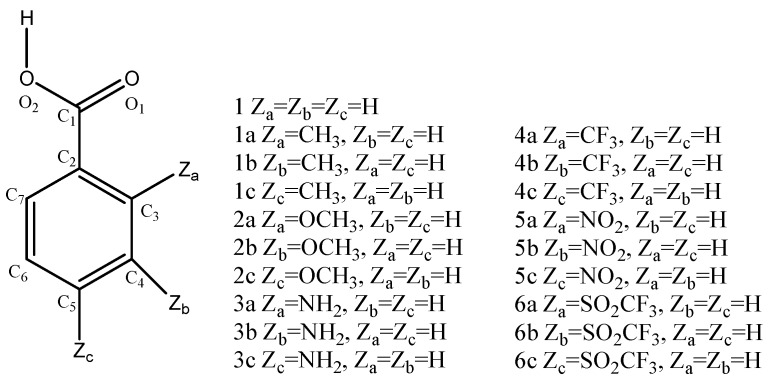
Substituted benzoic acids.

Most of the previous studies have evaluated the acidity of the *meta*- and *para*-substituted benzoic acids, while in general the *ortho*-substituted has been excluded. Therefore, in this paper we focused our attention in the effect of the substituent Z on the gas phase acidity of a set of *ortho*-, *meta*- and *para*-substituted benzoic acids ZC_6_H_4_COOH (**1a**–**6c**, Figure 1) in terms of the density functional theory descriptors electronegativity *χ* [21] and softness *S* (related with the inductive and polarizability effects, respectively [24,25,26,27,28]) of substituent (Z) and fragments (ZC_6_H_4_, ZC_6_H_4_COO). We found that there is a good relationship between the acidity of substituted benzoic acids and the electronegativity of ZC_6_H_4_, ZC_6_H_4_COO and ZC_6_H_4_COOH indicating the importance of the inductive contributions. While for the softness of ZC_6_H_4_COOH and its fragments there is not a relationship with the acidity, suggesting less importance of the polarizability contributions. The Fukui function, a density functional theory (DFT) descriptor related with the resonance effect [20,29], showed that the electron-releasing substituent attached to the phenyl ring of benzoic acid (**1a**–**3c**) decreases the acidity in the trend *orth*o > meta > *para***,** and the electron-withdrawing substituent (**4a**–**6c**) increases the acidity in the trend *ortho* < *meta* < *par*a.

## 2. Results and Discussion

**Scheme 1:**
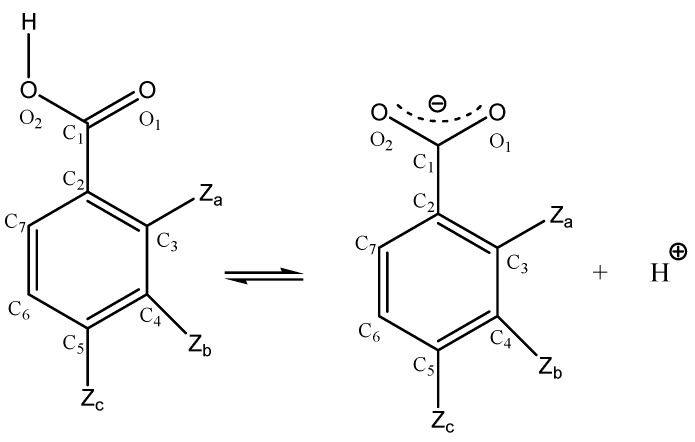
The ionization reactions for ZC_6_H_4_COOH.

Scheme 1 shows the ionization reaction in the gas phase for the substituted benzoic acids.

**Figure 2:**
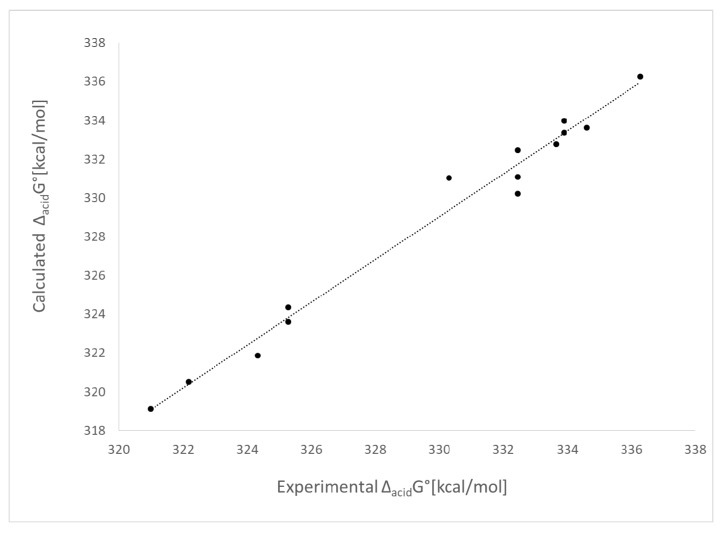
Calculated Δ_acid_G° vs experimental Δ_acid_G° values for a set of substituted benzoic acids. Gas phase acidity in kcal/mol, R^2^ = 0.98.

Table 1 shows our gas phase acidity values calculated at the B3LYP/6-311++G(2d,2p) level of theory using GAUSSIAN09 [30] for **1a**–**6c**, they were obtained as Δ_acid_*G*° = *G*°(anion) + *G*°(H^+^) − *G*°(acid). The calculated Δ_acid_*G*° values were close to the experimental values reported in the literature (see Table 1) [31]. Linear regression analysis shows that there is a good correlation between experimental and theoretical Δ_acid_*G*° values with R^2^ = 0.98 (Figure 2).

The calculated gas phase acidity values are consistent with the substituent electronic effect, being the benzoic acid with substituent NH_2_ (compounds **3a**–**3c**) and SO_2_CF_3_ (compounds **6a**–**6c**) the least and most acidic, respectively; they correspond to the two extremes for which δΔ_acid_*G*° = 19.37 kcal/mol. Interesting for the electron-releasing substituent attached to the phenyl ring the acidity follows the trend *ortho* > *meta* > *para*, and the average value for the two extremes is δΔ_acid_*G*° = 3.7 kcal/mol, while for electron-withdrawing substituent the acidity follows the trend *ortho* < *meta* < *para*, and the average value for the two extremes is δΔ_acid_*G*° = 2.4 kcal/mol.

Considering that the steric effects are of limited importance for acidity [3] and the usefulness of the electronegativity and polarizability [24,25,26,27,28], we study the electronic effect of the substituent Z in the acidity of the substituted benzoic acids using a two parameter linear model Δ_acid_*G*° = m*x* + b, where *x* is the electronegativity *χ* or softness *S* of Z, C_6_H_4_, ZC_6_H_4_, ZC_6_H_4_COO and ZC_6_H_4_COOH (we have observed that the model of two parameters is sufficient for cyclic derivatives [17], while for acyclic derivatives the best-fitting values were achieved for a triple-parameter equation [19]). The electronegativity and softness of ZC_6_H_4_COOH were obtained as the negative of the chemical potential *μ* and the inverse of the hardness *S* = 1/(2*η*), respectively, where *μ* = − (*I* + *A*)/2 = −*χ* and *η* = (*I*−*A*)/2, where *I* is the ionization potential and *A* the electron affinity [21]. The electronegativity and softness for the fragments Z, C_6_H_4_, ZC_6_H_4_ and ZC_6_H_4_COO were obtained using the condensed Fukui function for each fragment and the Hirshfeld charges analysis [32,33].

Table 2 shows the two-parameter correlation analysis for *S*_fragment_ and Δ_acid_*G*°, the poor correlation coefficient values obtained shows that there is not direct relationship between Δ_acid_*G*°, and the softness of Z, ZC_6_H_4_, ZC_6_H_4_COO and ZC_6_H_4_COOH. The nonlinearity suggesting that the polarizability contribution has not an important role in the acidity of substituted benzoic acids. Instead, Table 3 shows that there is a good linear correlation between *χ*_fragment_ and Δ_acid_*G*° for ZC_6_H_4_, ZC_6_H_4_COO and ZC_6_H_4_COOH. The acidity of the substituted benzoic acids increases when the electronegativity of the fragments ZC_6_H_4_, ZC_6_H_4_COO increases. The nonlinearity between the electronegativity of the substituent Z and Δ_acid_*G*° suggesting that there is no direct interaction between the substituent and the hydrogen atom of the carboxylic group. Therefore, the substituent Z transmits its inductive effect through the π system of the benzene ring C_6_H_4_ and the hydrogen atom of the carboxylic group shows the effect of the electronegativity of the whole fragment ZC_6_H_4_COO. This is consistent with the good relationship obtained between the hydrogen charge and the Δ_acid_*G*° (Table 2) [16].

**Figure 3:**
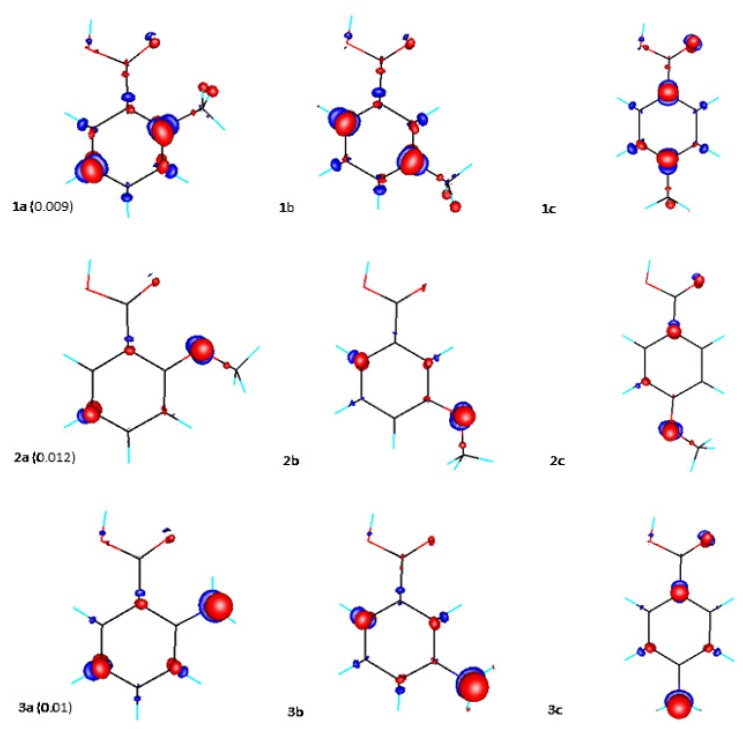
The surface plot of the electrophilic Fukui function $f^{-}(r)$ for the benzoic acids with electron-releasing substituent (**1a**–**3c**).

We have shown previously that the resonance hybrid structure can be described in terms of the Fukui function [19,29]. The Fukui function f(r) represents the change of the electron density ρ(r) in a given point with respect to the change in the number of electrons *N*, $f(r)={\left(\partial \rho (r)/\partial N\right)}_{\nu (r)}$ and the relationship between f(r) and the change of energy *E* of the system produced by the charge redistribution due to the loss or gain of an electron is obtained from f(r)=(∂(δE/δν)/∂N) [21]. The charge redistribution in the benzoic acid triggered by the substituent can be obtained from the Fukui function for electrophilic attack $f^{-}(r)=\rho_{N}(r)-\rho_{N-1}(r)$ (for loss an electron), and nucleophilic attack $f^{+}(r)=\rho_{N+1}(r)-\rho_{N}(r)$ (for gain an electron), where $\rho_{N}(r)$, $\rho_{N-1}(r)$ and $\rho_{N+1}(r)$ are the electron density of the species with N, N-1 and N+1 electrons, respectively [34]. Therefore, we considered $f^{-}(r)$ and $f^{+}(r)$ for the substituted benzoic acids with electron-releasing and electron-withdrawing substituent, respectively. Figure 3 shows that the largest value of $f^{-}(r)$ for **1a**–**3c** is located in general in the C atom opposite to the site where the substituent is located: the **C-6, C-7** and **C-2** atoms for the *ortho*-, *meta*- and *para*-position, respectively. Those atoms will be the most reactive because in the electronic redistribution they are the most suitable to give electron to an electrophile and according with the local HSAB principle [23] they will interact with soft electrophiles. As we can observe, these atoms will be the richest in electron density, and to the extend that they are further away from the carboxyl group they will interact less with it and allow the hydrogen atom to disassociate as a proton more easily. Therefore, the electron-releasing substituent (CH_3_, OCH_3_ and NH_2_) will increase the acidity of the benzoic acid in the trend *ortho-* > *meta-* > *para*-position.

**Figure 4:**
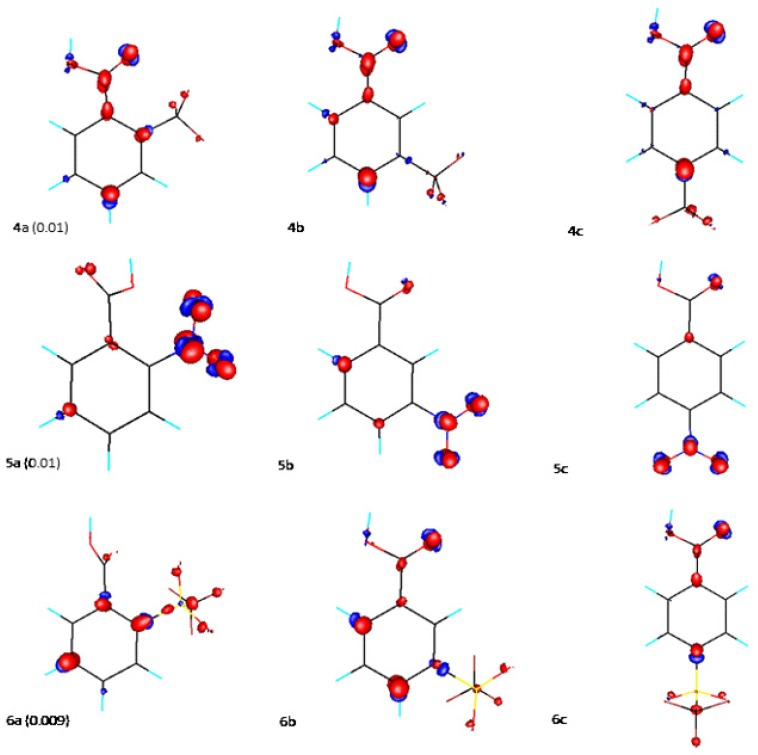
The surface plot of the nucleophilic Fukui function *f* ^+^ (**r**) for the benzoic acids with electron-withdrawing substituent (**4a**–**6c)**.

For the benzoic acid with the electron-withdrawing substituents (**4a**–**6c)** we examined the Fukui function for nucleophilic attack $f^{+}(r)$. Figure 4 shows that $f^{+}(r)$ is located in several atoms of the molecules **4a**–**6c**. As the substituent changes its position from *ortho* to *para*, the contribution of the C-2 atom increases. The C-2 atom will increase its reactivity because in the electronic redistribution it is suitable to gain electron to a nucleophile and according with the local HSAB principle [23] it will interact with soft nucleophiles. The C-2 atom will be poor in electron density, and it will interact with the carboxyl group and allow the hydrogen atom to disassociate as a proton more easily. Therefore, the electron-releasing substituent (CF_3_, NO_2_ and CF_3_SO_2_) will increase the acidity of the benzoic acid in the trend *para-* > *meta-* > *ortho*-position.

The previous results suggest that the inductive and resonance effects are more important than the polarizability effects in the acidity of substituted benzoic acids, and the inductive and resonance effects (IE and RE, respectively) of the substituent can be approximated as the difference of the gas phase acidity values between the two extremes: IE(*ortho*) = Δ_acid_*G*°*_ortho_*(-CH_3_) − Δ_acid_*G*°*_orth_*_o_(-SO_2_CF_3_), IE(*meta*) = Δ_acid_*G*°*_meta_*(-CH_3_) − Δ_acid_*G*°*_met_*_a_(-SO_2_CF_3_), IE(*para*)Δ_acid_*G*°*_para_*(-CH_3_) − Δ_acid_*G*°*_para_*(-SO_2_CF_3_), RE(CH_3_)= Δ_acid_G°*_para_*(-CH_3_) − Δ_acid_*G*°*_ortho_*(-CH_3_), …, RE(-SO_2_CF_3_) = Δ_acid_G°*_ortho_*(-SO_2_CF_3_) − Δ_acid_G°*_para_*(-SO_2_CF_3_). With the calculated absolute gas phase acidity (Table 1) we can obtain the contribution of the resonance and inductive effects for **1a**–**6c**. Table 4 shows that in general the percentage contributions of the inductive effect is higher than the resonance effect. As we expected, the highest inductive and resonance effects are for the -SO_2_CF_3_ and -NH_2_ substituents in the *para*- and *ortho*-position, respectively.

## 3. Methodology and Computational Details

The ground state structures, energies and gas phase acidities of the *ortho-, meta- and para*-substituted benzoic acids **1a**–**6c** were calculated at the B3LYP/6-311++G(2d,2p) level of theory using GAUSSIAN09 [30]. Please find the information of Computed Cartesian Coordinates and Energies of Substituted Benzoic Acids in the Supplementary Materials. The default SCF = TIGHT convergence was used: convergence on RMS density matrix = 1.00D-08 within 64 cycles, convergence on MAX density matrix = 1.00D-06, and convergence on energy = 1.00D-06. The absolute gas-phase acidity Δ_acid_*G*° was obtained using Δ_acid_*G*° = *G*°(anion) + *G*°(H^+^) − *G*°(acid). The Gibbs free energy of the proton in the gas phase *G*°(H^+^) = −6.26 kcal/mol was obtained by means of partition functions using statistical thermodynamic relationships [35]. All of thermodynamics parameters were obtained at T = 298.15K and P = 1atm. The Fukui functions for electrophilic and nucleophilic attacks of **1a**–**6c** were visualized by the gOpenmol software [36]. Frequency calculations were carried out without any symmetry constraints to confirm that the structures obtained correspond to energy minima. In order to corroborate the quality of the B3LYP functional we made calculations of the gas phase acidity using MP/6-311++G(2d,2p) and the M062X/6-311++G(2d,2p) levels of theory. The agreement between the calculated and experimental gas phase acidity values of benzoic acids was −3.14 to +6.97 kcal/mol for M062X, −2.70 to 0.04 for MP2 and −2.60 to 1.99 kcal/mol for B3LYP, the experimental uncertainty was −2.01 to 2.01 kcal/mol, therefore B3LYP was very good within the expected experimental uncertainty. We characterized the conformational space of the species and found that conformers with the O=C-O-H dihedral close to zero give better gas phase acidity values than the O=C-O-H dihedral close to 180°. For example the gas phase acidity for *ortho*-amino substituent with the O=C-O-H dihedral near to 180° was 322.51 kcal/mol and for 0° was 331.04 kcal/mol, respectively; the experimental value is 330.31 ± 2.01 kcal/mol. The conformational analysis showed no disagreement in the DFT descriptors due to the change of the dihedral angle O=C-O-H from 0 to 180 degrees; we observed that the linear relations of free energy between acidity and the DFT descriptors do not depend on the conformational peculiarities of this molecule. We compared the electrophilic and nucleophilic Parr function [37] for the *ortho-, meta- and para*-substituted benzoic acids **1a**–**6c** and we obtained not difference with the Yang and Mortier Fukui functions.

## 4. Conclusions

The electronegativity and the Fukui function are good descriptors to account for substituent effects in the gas phase acidity of a set of *ortho*-, *meta* and *para*-substituted benzoic acids. The analysis of inductive and resonance effects of the substituent in the acidity of substituted benzoic acids shows that they contribute in the opposite way, for electron-withdrawing substituent the acidity increases from *ortho*- to *para*-isomers when the inductive and resonance effects increases and decreases, respectively, while for electron-releasing substituent the acidity increases from *para*- to *ortho*-isomers when the inductive and resonance effects decreases and increases, respectively. The highest inductive and resonance effects are for the -SO_2_CF_3_ (electron-withdrawing) and -NH_2_ (electron-releasing) substituents in the *para*- and *ortho*-position, respectively. The acidity difference between the *para*-SO_2_CF_3_ and *ortho*-NH_2_ benzoic acids is 14.16 kcal/mol. Recently, Georgousaki et al. [38] have pointed out the great importance of substituted benzoic acids in biology. They suggested that the *p*-hydroxybenzoic acid can be considered as a promising candidate for the development of novel modulators of the proteostasis network, and likely of anti-aging agents. They showed that benzoic acid derivatives substituted with OH, CH_3_ and Cl enhance the activity of the two main cell protein degradation systems (the ubiquitin-proteasome and the autophagy-lysosome pathway) and especially the activity of cathepsins B and L. We hope that our results will open up the possibility of analyzing the chemical-biological interactions of substituted benzoic acids in terms of DFT reactivity descriptors.

## Figures and Tables

**Table 1:** Absolute gas phase acidities for **1a**–**6c**. The values are reported in kcal/mol. Δ_acid_*G*° = *G*°(anion) + G°(H^+^) − *G*°(acid). The experimental values are given in parentheses and were obtained from http://webbook.nist.gov/chemistry/ [31].

	1	2	3	4	5	6
**a**	331.09 (332.46 ± 2.01)	330.21 (332.46 ± 2.01)	331.03 (330.31 ± 2.01)	325.99 (-------)	321.86 (324.33 ± 2.01)	319.06 (-----)
**b**	332.76 (333.65 ± 2.01)	332.47 (332.46 ± 2.01)	333.62 (334.61 ± 2.01)	324.34 (325.29 ± 2.01)	320.51 (322.18 ± 2.01)	318.19 (-----)
**c**	333.35 (333.89 ± 2.01)	333.97 (333.89 ± 2.01)	336.24 (336.28 ± 2.01)	323.61 (325.29 ± 2.01)	319.13 (320.98 ± 2.01)	316.87 (-----)

**Table 2:** Two parameter correlation analysis Δ_acid_*G*° (kcal/mol) = b + m*S*_fragment_ (eV^-1^) for **1a**–**6c**.

Fragment	Position	R^2^
Z	*o*	0.1974
	*m*	0.0004
	*p*	0.0493
C_6_H_4_	*o*	0.5038
	*m*	0.855
	*p*	0.8021
ZC_6_H_4_	*o*	0.0011
	*m*	0.4267
	*p*	0.227
ZC_6_H_4_COO	*o*	0.0089
	*m*	0.31
	*p*	0.0026
ZC_6_H_4_COOH	*o*	0.0034
	*m*	0.2933
	*p*	0.0008
qH	*o*	0.9239
	*m*	0.9938
	*p*	0.9903

**Table 3:** Two parameter correlation analysis Δ_acid_*G*° (kcal/mol) = b + m*χ*_fragment_ (eV) for **1a**–**6c**.

Fragment	Position	R^2^
Z	*o*	0.7333
	*m*	0.5409
	*p*	0.0125
C_6_H_4_	*o*	0.4642
	*m*	0.6352
	*p*	0.6654
ZC_6_H_4_	*o*	0.9415
	*m*	0.8498
	*p*	0.8699
ZC_6_H_4_COO	*o*	0.8422
	*m*	0.920
	*p*	0.9746
ZC_6_H_4_COOH	*o*	0.8266
	*m*	0.9206
	*p*	0.9711

**Table 4:** Percentage contribution of resonance and inductive effects for the substituted benzoic acids. The percentage for the inductive effect is given in parentheses.

	1	2	3	4	5	6
a	18.79% (81.21%)	31.26% (68.74%)	43.31% (56.69%)	19.78% (80.22%)	22.69% (77.31%)	18.20% (81.80%)
b	15.51% (84.49%)	25.80% (74.20%)	35.76% (64.24%)	16.33% (83.67%)	18.74% (81.26%)	15.03% (84.97%)
c	13.71% (86.29%)	22.82% (77.18%)	31.61% (68.39%)	14.44% (85.56%)	16.57% (83.43%)	13.29% (86.71%)

## Supplementary Materials

Supplementary materials can be accessed at, Computed Cartesian Coordinates and Energies of Substituted Benzoic Acids (Page 2S), Computed Hirshfeld Atomic Charges (Page 18S), Electronegativity and Global Softness (Page 24S), Condensed Fukui Function Calculated Using Hirshfeld Atomic Charges (Page 25S), Absolute gas phase acidities for **1a**–**6c** calculated at the M062X/6-311++G(2d,2p) and MP2/6-311++G(2d,2p) levels of theory (Page 29S).
