# Supplementary material for: Analysis of the Gas Phase Acidity of Substituted Benzoic Acids Using Density Functional Concepts
Source: Molecules. 2020 Apr 2;25(7):1631. doi: 10.3390/molecules25071631 (PMC7180886; doi:10.3390/molecules25071631)
Supplement: Supplementary file 1 [file molecules-25-01631-s001.pdf]

Supplementary Information

for

Analysis of the gas phase acidity of substituted benzoic acids using density functional concepts

Jorge A. Amador-Balderas[1], Michael-Adán Martínez-Sánchez[2], Ramsés E. Ramírez[1]*, Francisco Méndez[2,3,4]* and Francisco J. Meléndez[5]

1 Departamento de Fisicomatemáticas, Facultad de Ciencias Químicas, Av. San Claudio y 14 Sur, Col. San Manuel, Benemérita Universidad Autónoma de Puebla, C.P. 72570, Puebla, Pue., México.
2 Departamento de Química, División de Ciencias Básicas e Ingeniería, Universidad Autónoma Metropolitana-Iztapalapa, A.P. 55-534, México, D.F., 09340 México.
3 LE STUDIUM GUEST RESEARCH FELLOW, Loire Valley Institute for Advanced Studies, 45000 Orléans & Tours, France.
4 Conditions Extrêmes et Matériaux: Haute Température et Irradiation (CEMHTI), UPR3079 CNRS, CEMHTI 1, Avenue de la Recherche Scientifique, 45071 Orléans, France.
5 Departamento de Fisicoquímica, Facultad de Ciencias Químicas, Av. San Claudio y 14 Sur, Col. San Manuel, Benemérita Universidad Autónoma de Puebla, C.P. 72570, Puebla, Pue., México.

*fm@xanum.uam.mx and ramses.ramirez@correo.buap.mx

Table of contents

Computed Cartesian Coordinates and Energies of Substituted Benzoic Acids.................... 2S

Computed Hirshfeld Atomic Charges 18S

Electronegativity and Global Softness 24S

Condensed Fukui Function Calculated Using Hirshfeld Atomic Charges 25S

Absolute gas phase acidities for **1a–6c** calculated at the M062X/6-311++G(2d,2p) and MP2/6-311++G(2d,2p)levels of theory ……… …………………..................................... 29S

All data were obtained at the B3LYP/6-311++G(2d,2p) level of theory.

Computed Cartesian Coordinates and Energies of substituted benzoic acids (in Hartrees)

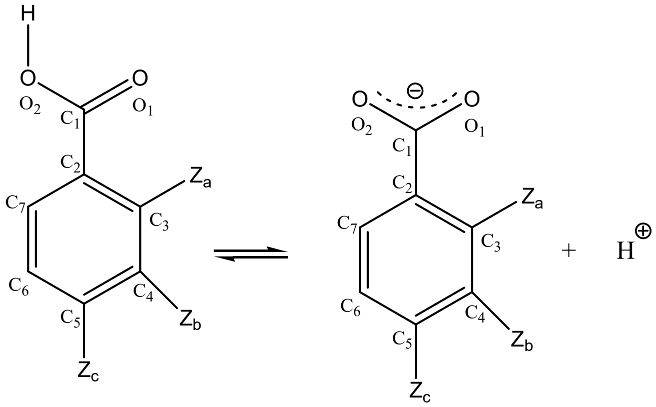

Benzoic acid   Benzoate anion

1 Z_a=Z_b=Z_c=H	
1a Z_a=CH_3, Z_b=Z_c=H	4a Z_a=CF_3, Z_b=Z_c=H
1b Z_b=CH_3, Z_a=Z_c=H	4b Z_b=CF_3, Z_a=Z_c=H
1c Z_c=CH_3, Z_a=Z_b=H	4c Z_c=CF_3, Z_a=Z_b=H
2a Z_a=OCH_3, Z_b=Z_c=H	5a Z_a=NO_2, Z_b=Z_c=H
2b Z_b=OCH_3, Z_a=Z_c=H	5b Z_b=NO_2, Z_a=Z_c=H
2c Z_c=OCH_3, Z_a=Z_b=H	5c Z_c=NO_2, Z_a=Z_b=H
3a Z_a=NH_2, Z_b=Z_c=H	6a Z_a=SO_2CF_3, Z_b=Z_c=H
3b Z_b=NH_2, Z_a=Z_c=H	6b Z_b=SO_2CF_3, Z_a=Z_c=H
3c Z_c=NH_2, Z_a=Z_b=H	6c Z_c=SO_2CF_3, Z_a=Z_b=H

Acid 1a

```
Sum of electronic and thermal Free energies = -460.179703 a.u.
E (RB3LYP) = -460.28867884 a.u.
Sum of electronic and thermal Enthalpies = -460.135976 a.u.
```

C	-0.19857600	-0.23298600	-0.00002900
C	0.47075300	-1.46460600	-0.00002200
C	1.85454300	-1.52820900	0.00000000
C	2.58818500	-0.34764000	0.00001800
C	1.93218900	0.87646600	0.00001300
C	0.53863200	0.97248300	-0.00000800
H	-0.10961200	-2.37364800	-0.00003700
H	2.35371700	-2.48688700	0.00000400
H	3.66950700	-0.37737200	0.00003600
H	2.51294300	1.78917700	0.00002700
C	-1.68385000	-0.22121800	-0.00005600
O	-2.39178800	0.75969300	0.00003400
O	-2.22793100	-1.46983700	0.00002700
H	-3.18597700	-1.33918600	0.00008100
C	-0.08824100	2.34252900	-0.00001100

H	-0.72668900	2.49139000	-0.86946000
H	-0.72670900	2.49138500	0.86942300
H	0.68875700	3.10536200	0.00000000

Acid 1b

Sum of electronic and thermal Free energies = -460.184648 a.u.
E (RB3LYP) = -460.29222707 a.u.
Sum of electronic and thermal Enthalpies = -460.139662 a.u.

C	-0.58532600	0.10378600	-0.00001200
C	-0.31393900	1.47215500	0.00000600
C	1.00713500	1.90360000	-0.00000500
C	2.04548100	0.98031900	-0.00002600
C	1.79145300	-0.39588000	-0.00002800
C	0.46619600	-0.81720900	-0.00003100
H	-1.12516100	2.18358100	0.00001100
H	1.22773700	2.96215100	-0.00001000
H	3.07004700	1.33113200	-0.00005000
H	0.22482100	-1.87090700	-0.00005900
C	-1.97278400	-0.42532500	-0.00002900
O	-2.26934500	-1.59677100	-0.00002800
O	-2.91848100	0.54986000	0.00005700
H	-3.77319900	0.09736400	0.00007300
C	2.92766100	-1.38764200	0.00004800
H	3.56165100	-1.26038300	0.87923500
H	2.55842500	-2.41151000	-0.00104100
H	3.56303200	-1.25897300	-0.87792500

Acid 1c

Sum of electronic and thermal Free energies = -460.185856 a.u.
E (RB3LYP) = -460.29288638 a.u.
Sum of electronic and thermal Enthalpies = -460.140338 a.u.

C	0.70140900	0.03388800	-0.00034300
C	0.01781900	-1.18363600	-0.00442800
C	-1.37022600	-1.20207400	-0.00975000
C	-2.10846700	-0.01638600	-0.00938100
C	-1.41211400	1.19698200	-0.00938000
C	-0.02715900	1.22603300	-0.00386300
H	0.57272300	-2.10950500	-0.00641500
H	-1.88882200	-2.15192600	-0.01705800
H	-1.96452500	2.12771700	-0.01643100
H	0.50892700	2.16392500	-0.00527400
C	2.18122000	0.11337800	0.00293500
O	2.82230000	1.13814800	0.00461600
O	2.78426500	-1.10478000	0.00466300
H	3.73616900	-0.93422700	0.00729000
C	-3.61444500	-0.04001300	0.01407000
H	-4.03361200	0.77779000	-0.57155100
H	-4.00448000	-0.97757100	-0.37949700
H	-3.98712300	0.06782500	1.03554600

Acid 2a

Sum of electronic and thermal Free energies = -535.404842 a.u.
E (RB3LYP) = -535.51534497 a.u.
Sum of electronic and thermal Enthalpies = -535.357000 a.u.

C	0.64820300	1.70568200	-0.00004400

C	-0.47280900	2.52045200	-0.00002100
C	-1.73233100	1.93394500	0.00003900
C	-1.87012100	0.55259400	0.00002700
C	-0.74295100	-0.27689200	-0.00005600
C	0.54704400	0.30974500	-0.00004900
H	1.63253100	2.14585300	-0.00004800
H	-0.36480100	3.59526200	-0.00005700
H	-2.62048300	2.55150500	0.00009800
H	-2.85800600	0.12148200	0.00009700
C	1.78087700	-0.51572500	-0.00000400
O	1.85361000	-1.71961600	-0.00003200
O	2.91367400	0.25122700	0.00010700
H	3.64848700	-0.37681800	0.00012700
O	-0.82654300	-1.62193500	-0.00015100
C	-2.10729800	-2.23948600	0.00009600
H	-1.91176700	-3.30722200	0.00007400
H	-2.67761600	-1.97467000	0.89273300
H	-2.67795500	-1.97469900	-0.89233400

Acid 2b

Sum of electronic and thermal Free energies = -535.410485 a.u.
E (RB3LYP) = -535.52247645 a.u.
Sum of electronic and thermal Enthalpies = -535.364174 a.u.

C	-0.83616700	1.49314600	-0.00000200
C	0.44545800	2.01848200	-0.00001000
C	1.56419100	1.18617300	-0.00000800
C	1.39381500	-0.19944400	0.00000100
C	0.10708100	-0.73695800	0.00000600
C	-1.00056400	0.10281400	0.00000500
H	-1.69856500	2.14044900	-0.00000200
H	0.58756200	3.09035500	-0.00001700
H	2.55026300	1.62390100	-0.00001400
H	-0.02673800	-1.80826200	0.00001000
C	-2.34523000	-0.53196800	0.00001000
O	-2.55166300	-1.72186700	-0.00002600
O	-3.36052300	0.36978000	0.00001800
H	-4.17920100	-0.14511500	0.00000100
O	2.41304700	-1.10325800	0.00000400
C	3.74871800	-0.61795400	0.00000500
H	4.38358200	-1.49873700	0.00001400
H	3.95620100	-0.02279700	-0.89212600
H	3.95619500	-0.02278300	0.89212800

Acid 2c

Sum of electronic and thermal Free energies = -535.413393 a.u.
E (RB3LYP) = -535.52549207 a.u.
Sum of electronic and thermal Enthalpies = -535.367086 a.u.

C	0.54058600	1.25960600	0.00000100
C	-0.83136700	1.41244800	0.00000000
C	-1.66434700	0.28671600	-0.00001200
C	-1.10332900	-0.99391000	-0.00002500
C	0.27707500	-1.13470800	-0.00002200
C	1.11390100	-0.01962500	-0.00000900
H	1.17798200	2.13063500	0.00001100
H	-1.28602600	2.39249800	0.00000900
H	-1.72618600	-1.87433100	-0.00003900

H	0.72346800	-2.11853800	-0.00003100
C	2.57512600	-0.23606300	-0.00000500
O	3.12165900	-1.31549200	0.00002400
O	3.28938200	0.92204400	0.00000500
H	4.22082000	0.66253500	0.00002700
O	-2.99708300	0.54055600	-0.00001800
C	-3.90278300	-0.55793100	0.00003600
H	-3.77723600	-1.17415500	0.89227600
H	-4.89633100	-0.12054600	0.00008200
H	-3.77732500	-1.17417000	-0.89220700

Acid 3a

Sum of electronic and thermal Free energies = -476.248501 a.u.
E (RB3LYP) = -476.34729293 a.u.
Sum of electronic and thermal Enthalpies = -476.205831 a.u.

C	0.21226700	-0.22907200	-0.01037500
C	-0.47144200	-1.45783900	-0.00699000
C	-1.84903600	-1.52015300	0.00426400
C	-2.57903100	-0.32565400	0.01416800
C	-1.93903300	0.89487900	0.00660100
C	-0.53143900	0.98300700	-0.01059400
H	0.11114400	-2.36606700	-0.00917200
H	-2.35520300	-2.47415900	0.00799200
H	-3.66053200	-0.35463000	0.02638800
H	-2.51569500	1.81086500	0.00743000
C	1.67682600	-0.19716400	0.00421000
O	2.37165800	0.80624600	0.03625600
O	2.25728800	-1.42509700	-0.01459700
H	3.21107400	-1.26774000	0.00269400
N	0.06813700	2.20588600	-0.05608200
H	-0.48319000	3.02458400	0.12173800
H	1.06920400	2.24872200	0.05452400

Acid 3b

Sum of electronic and thermal Free energies = -476.243215 a.u.
E (RB3LYP) = -476.34147946 a.u.
Sum of electronic and thermal Enthalpies = -476.200014 a.u.

C	-0.56589000	0.10773300	-0.00182000
C	-0.26862400	1.47235500	0.00500900
C	1.06386400	1.86695600	0.00595400
C	2.08502500	0.92689800	-0.00132600
C	1.79383900	-0.44423400	-0.00761400
C	0.45546900	-0.84039400	-0.00823200
H	-1.06243100	2.20194100	0.01070600
H	1.31275000	2.91923700	0.01239000
H	3.11777000	1.25202200	-0.00690300
H	0.19003700	-1.88854700	-0.01651400
C	-1.96458200	-0.39383200	0.00133000
O	-2.28471500	-1.55944500	0.00740100
O	-2.89084800	0.59889600	-0.00247300
H	-3.75386800	0.16252500	0.00000300
N	2.81763500	-1.39000600	-0.07504600
H	3.70925800	-1.09122200	0.28530500
H	2.57293800	-2.31440400	0.24110700

Acid 3c

Sum of electronic and thermal Free energies = -476.246443 a.u.
E (RB3LYP) = -476.34478458 a.u.
Sum of electronic and thermal Enthalpies = -476.203329 a.u.

C	0.68645400	0.03483600	-0.00163800
C	-0.00430300	-1.18213300	-0.00283100
C	-1.38666800	-1.21010400	-0.00486000
C	-2.12440500	-0.01629800	-0.00503300
C	-1.43021200	1.20453300	-0.00562300
C	-0.04965100	1.22491800	-0.00331900
H	0.54930000	-2.10891000	-0.00082100
H	-1.90751100	-2.15889300	-0.00979400
H	-1.98530200	2.13370900	-0.01107300
H	0.48412600	2.16425900	-0.00153600
C	2.15759300	0.11452300	0.00279300
O	2.80451500	1.13810300	0.00596300
O	2.76451000	-1.10554000	0.00338100
H	3.71503100	-0.92913700	0.00584900
N	-3.50784500	-0.04082100	-0.05900700
H	-3.95826900	-0.88931200	0.23985400
H	-3.98751100	0.79186700	0.23888700

Acid 4a

Sum of electronic and thermal Free energies = -758.021258 a.u.
E (RB3LYP) = -758.10565905 a.u.
Sum of electronic and thermal Enthalpies = -757.975232 a.u.

C	-0.92277600	0.20889400	-0.00011300
C	-2.18613500	-0.38987100	-0.00010000
C	-2.33646500	-1.76955800	-0.00001500
C	-1.21179400	-2.57831100	0.00007100
C	0.05377900	-2.00225600	0.00006300
C	0.22031800	-0.62090700	-0.00003400
H	-3.05815500	0.24330500	-0.00015400
H	-3.32656200	-2.20299700	-0.00000700
H	-1.30814200	-3.65496500	0.00014400
H	0.92530200	-2.63594000	0.00012400
C	-0.84984200	1.70364600	-0.00017300
O	0.15120300	2.36961900	0.00021400
O	-2.08330000	2.27879800	0.00014100
H	-1.92927900	3.23359300	0.00038400
C	1.65045700	-0.10069600	-0.00004700
F	1.93490500	0.62786800	1.09159000
F	1.93502100	0.62751800	-1.09188600
F	2.53544700	-1.13049400	0.00015900

Acid 4b

Sum of electronic and thermal Free energies = -758.034600 a.u.
E (RB3LYP) = -758.11796778 a.u.
Sum of electronic and thermal Enthalpies = -757.987517 a.u.

C	-1.49964500	0.15274900	-0.00006200
C	-1.56180400	1.54593500	-0.00009200
C	-0.38890100	2.29298100	-0.00016000
C	0.84305500	1.65486100	-0.00018900
C	0.90350500	0.26026600	-0.00017700

C	-0.26044800	-0.49156100	-0.00011400
H	-2.52215300	2.03816200	-0.00006600
H	-0.43639000	3.37250700	-0.00019700
H	1.75597600	2.23352300	-0.00025000
H	-0.22967800	-1.57006500	-0.00010600
C	-2.72210600	-0.69545700	0.00004800
O	-2.72148800	-1.90187800	0.00004600
O	-3.86882200	0.02774600	0.00011100
H	-4.59633600	-0.60991200	0.00017600
C	2.25618800	-0.39925800	0.00002300
F	2.98325800	-0.04159000	1.08423400
F	2.17967500	-1.74140600	-0.00188100
F	2.98506700	-0.03858800	-1.08196000

Acid 4c

Sum of electronic and thermal Free energies = -758.037065 a.u.
E (RB3LYP) = -758.11787879 a.u.
Sum of electronic and thermal Enthalpies = -757.986438 a.u.

C	-1.71882800	-0.03202900	-0.00445200
C	-1.04079500	1.18685100	-0.00984700
C	0.34810000	1.21009600	-0.01856000
C	1.05833200	0.01466800	-0.02321000
C	0.38576100	-1.20716900	-0.01780900
C	-0.99860000	-1.22895100	-0.00885700
H	-1.59690900	2.11140200	-0.00852900
H	0.87494000	2.15195800	-0.02611000
H	0.94359300	-2.13227000	-0.02579600
H	-1.53908500	-2.16375900	-0.00673600
C	-3.20532400	-0.11067900	0.00442900
O	-3.83802900	-1.13809500	0.00787100
O	-3.80123300	1.10643200	0.00887500
H	-4.75545400	0.94700000	0.01493100
C	2.56382300	0.01657200	0.00029300
F	3.04101100	-0.33391600	1.21754400
F	3.08048900	-0.86426200	-0.88323800
F	3.08207900	1.22515800	-0.29137900

Acid 5a

Sum of electronic and thermal Free energies = -625.435630 a.u.
E (RB3LYP) = -625.51653385 a.u.
Sum of electronic and thermal Enthalpies = -625.388516 a.u.

C	-0.05614600	-0.73068800	-0.00114400
C	-1.05768400	-1.69911400	0.01470000
C	-2.39950200	-1.33708100	0.02959900
C	-2.76034000	0.00542100	0.03088600
C	-1.77797800	0.98692500	0.00499300
C	-0.44400600	0.61025100	-0.02232600
H	-0.76754900	-2.73959200	0.02336500
H	-3.16055500	-2.10426000	0.04322400
H	-3.80219400	0.29179200	0.04062400
H	-2.02815100	2.03633800	-0.01279500
C	1.36108300	-1.21474400	0.05926100
O	1.78756500	-2.11545900	-0.61403500
O	2.07368200	-0.59571800	1.02094200

H	2.97663800	-0.94312300	0.98123700
N	0.55496800	1.68621900	-0.16856500
O	0.31651400	2.75399700	0.37950100
O	1.53529800	1.44836800	-0.86034800

Acid 5b

Sum of electronic and thermal Free energies = -625.447520 a.u.
E (RB3LYP) = -625.52859207 a.u.
Sum of electronic and thermal Enthalpies = -625.400382 a.u.

C	-1.16422000	0.15156400	-0.00006000
C	-1.14567400	1.54864300	-0.00003100
C	0.06526800	2.23208200	0.00000200
C	1.26400000	1.53160700	0.00000800
C	1.22457800	0.14234000	-0.00001900
C	0.03315000	-0.56171100	-0.00005100
H	-2.07751300	2.09297700	-0.00003800
H	0.07605400	3.31235300	0.00002300
H	2.21715100	2.03614900	0.00003400
H	0.02784100	-1.63992800	-0.00007100
C	-2.43438000	-0.62679000	-0.00010600
O	-2.49955900	-1.83027000	0.00003200
O	-3.53505100	0.16304600	0.00008500
H	-4.30095500	-0.42806500	0.00019200
N	2.49845500	-0.60770300	-0.00001200
O	2.43747000	-1.82912900	0.00000900
O	3.53612800	0.04310600	0.00005900

Acid 5c

Sum of electronic and thermal Free energies = -625.447559 a.u.
E (RB3LYP) = -625.52860492 a.u.
Sum of electronic and thermal Enthalpies = -625.400350 a.u.

C	-1.35212800	0.03427000	-0.00001300
C	-0.67091100	-1.18533700	-0.00000700
C	0.71612400	-1.20690700	0.00001100
C	1.40062100	0.00036900	0.00002300
C	0.74582900	1.22518900	0.00001800
C	-0.63969300	1.23558000	0.00000100
H	-1.22500800	-2.11086400	-0.00001800
H	1.26707100	-2.13363400	0.00001500
H	1.31914900	2.13823800	0.00002800
H	-1.18529000	2.16726000	-0.00000400
C	-2.84136900	0.10738200	-0.00003500
O	-3.47447900	1.13366200	0.00000300
O	-3.43012000	-1.11151700	0.00001000
H	-4.38582800	-0.96002300	0.00003200
N	2.87999700	-0.01850500	0.00004400
O	3.45965500	1.05945300	-0.00003100
O	3.43233000	-1.11093800	-0.00002400

Acid 6a

Sum of electronic and thermal Free energies = -1306.667309 a.u.
E (RB3LYP) = -1306.75303312 a.u.
Sum of electronic and thermal Enthalpies = -1306.608470 a.u.

C	-1.62402400	0.24727500	-0.09513900
C	-2.85615900	-0.28560700	-0.46975600

C	-3.05745400	-1.66035600	-0.49762600
C	-2.03468800	-2.52754100	-0.13214000
C	-0.80817500	-2.01469000	0.26518400
C	-0.60548600	-0.63662600	0.27528200
H	-3.65886600	0.38408300	-0.74360300
H	-4.01911800	-2.05195500	-0.79828200
H	-2.19235000	-3.59633000	-0.14260700
H	-0.01269100	-2.66841000	0.58859200
C	-1.46888600	1.74337200	-0.17615400
O	-0.89923000	2.31336900	-1.06559100
O	-2.13602200	2.36506000	0.81431700
H	-2.00622400	3.31784900	0.69289200
S	0.97761600	-0.06347900	0.88749900
O	0.86729800	1.31557500	1.30269300
O	1.54042100	-1.08643700	1.73981200
C	2.08127300	-0.07561800	-0.65374000
F	1.62161500	0.75229900	-1.58163300
F	2.13153500	-1.31352100	-1.15756200
F	3.30343300	0.29773900	-0.28550200

Acid 6b

Sum of electronic and thermal Free energies = -1306.681221 a.u.
E (RB3LYP) = -1306.76757891 a.u.
Sum of electronic and thermal Enthalpies = -1306.622730 a.u.

C	-2.32544700	0.12346000	0.00618000
C	-2.53018700	1.45711300	0.36818400
C	-1.47356400	2.36071100	0.35345300
C	-0.20286400	1.94141400	-0.01750800
C	-0.01159500	0.60750300	-0.37171000
C	-1.05402900	-0.30794900	-0.36887500
H	-3.51821800	1.78272800	0.65563800
H	-1.64047700	3.39240900	0.62721400
H	0.62728700	2.63108200	-0.04885200
H	-0.89919400	-1.33517800	-0.66257400
C	-3.42891200	-0.87792500	-0.00035500
O	-3.29683100	-2.03733800	-0.30101600
O	-4.61934000	-0.35078100	0.37240900
H	-5.26567000	-1.07009200	0.33749500
S	1.61617400	0.06995100	-0.86666500
O	1.50799600	-1.12372300	-1.67217300
O	2.39527200	1.22002600	-1.26629200
C	2.36930100	-0.49677000	0.77507500
F	1.63845000	-1.47882200	1.30542400
F	2.41321500	0.52247600	1.63744400
F	3.60216800	-0.93598500	0.54909100

Acid 6c

Sum of electronic and thermal Free energies = -1306.681348 a.u.
E (RB3LYP) = -1306.76737261 a.u.
Sum of electronic and thermal Enthalpies = -1306.622545 a.u.

C	-2.62132300	0.03488500	-0.02955200
C	-1.95067500	-1.18212200	-0.16925300
C	-0.58455800	-1.19640000	-0.41382800
C	0.09183100	0.01485700	-0.51530200
C	-0.56169400	1.23718200	-0.38459200
C	-1.92613600	1.24087000	-0.14000000

H	-2.49631500	-2.10941700	-0.09073800
H	-0.05188900	-2.12661800	-0.54042000
H	-0.01152300	2.15996600	-0.48876200
H	-2.46834300	2.16882800	-0.03632500
C	-4.08926400	0.10035800	0.23237400
O	-4.71309300	1.12384100	0.36080000
O	-4.66524900	-1.12099200	0.31401800
H	-5.60693300	-0.97614200	0.48354200
S	1.84519400	0.00108700	-0.85338700
O	2.21071600	-1.26817600	-1.43949400
O	2.23387400	1.27038200	-1.42393900
C	2.59254900	-0.01671300	0.88622100
F	2.21516200	1.07023900	1.56258900
F	2.18828500	-1.10117400	1.55136700
F	3.91628100	-0.03261600	0.77931000

Anion 1a

Sum of electronic and thermal Free energies = -459.640560 a.u.
E (RB3LYP) = -459.734719833 a.u.
Sum of electronic and thermal Enthalpies = -459.596284 a.u.

C	-0.23425000	-0.27442900	-0.01127400
C	0.51670900	-1.44928300	-0.10234600
C	1.90670300	-1.43564300	-0.13595000
C	2.57998700	-0.22160200	-0.04191400
C	1.84546700	0.95447400	0.07519100
C	0.44610700	0.95640700	0.07801200
H	-0.03204300	-2.38073200	-0.13005200
H	2.46017700	-2.36349800	-0.22164800
H	3.66288700	-0.18777800	-0.05204800
H	2.36895900	1.90047000	0.16390100
C	-1.77769500	-0.39971200	-0.02415900
O	-2.39605100	0.52883200	-0.59678400
O	-2.23282300	-1.43034400	0.52169700
C	-0.28137900	2.27398200	0.19192300
H	-0.86332300	2.47186500	-0.70652000
H	-1.00426000	2.25959500	1.00761300
H	0.42869800	3.08701100	0.36255200

Anion 1b

Sum of electronic and thermal Free energies = -459.645067 a.u.
E (RB3LYP) = -459.738075705 a.u.
Sum of electronic and thermal Enthalpies = -459.599636 a.u.

C	-0.64581200	0.11792600	0.00031200
C	-0.33874600	1.48020500	0.00340400
C	0.98513900	1.90498800	-0.00252500
C	2.02275000	0.97293400	-0.00843900
C	1.74075400	-0.39420000	-0.00773300
C	0.40363700	-0.79921700	-0.00665500
H	-1.16401100	2.17872000	0.00619300
H	1.21713500	2.96409400	-0.00589900
H	3.05368700	1.30994100	-0.01649800
H	0.13632600	-1.84864400	-0.01335900
C	-2.11998900	-0.36611700	0.00065800
O	-2.27812600	-1.60919900	-0.01146000

O	-2.98304000	0.54213200	0.01329700
C	2.85409500	-1.41680600	0.01270500
H	2.97765900	-1.85281900	1.00826200
H	2.64800500	-2.24077400	-0.67257500
H	3.80955300	-0.97224900	-0.27118100

Anion 1c

Sum of electronic and thermal Free energies = -459.644568 a.u.
E (RB3LYP) = -459.737899720 a.u.
Sum of electronic and thermal Enthalpies = -459.599439 a.u.

C	-0.76346800	-0.00001100	-0.00001600
C	-0.04340800	1.19461100	-0.00481000
C	1.34882700	1.19612300	-0.01107800
C	2.07068700	-0.00000800	-0.01095400
C	1.34883800	-1.19612600	-0.01138000
C	-0.04341100	-1.19461900	-0.00506400
H	-0.60944600	2.11626900	-0.00745800
H	1.88560600	2.13952600	-0.01919500
H	1.88559900	-2.13953800	-0.01975300
H	-0.60941300	-2.11629800	-0.00782700
C	-2.31325700	-0.00000200	0.00416900
O	-2.85067100	-1.13215100	0.00623500
O	-2.85064800	1.13216800	0.00557700
C	3.58108700	0.00001000	0.01802000
H	3.98906800	-0.88211300	-0.47860800
H	3.98902600	0.88245300	-0.47806900
H	3.96473900	-0.00029900	1.04309500

Anion 2a

Sum of electronic and thermal Free energies = -534.862083 a.u.
E (RB3LYP) = -534.958256800 a.u.
Sum of electronic and thermal Enthalpies = -534.814279 a.u.

C	-0.05787500	-1.85353900	0.00007900
C	1.28660300	-2.22791700	0.00020300
C	2.26859400	-1.24784300	0.00013200
C	1.90239900	0.10034300	0.00000100
C	0.55383300	0.45716600	-0.00002700
C	-0.45493200	-0.52020800	0.00001200
H	-0.82748600	-2.61515500	-0.00006100
H	1.55960900	-3.27609600	0.00031900
H	3.31772700	-1.51607000	0.00017200
H	2.67478700	0.85565700	-0.00013500
C	-1.95339000	-0.15633200	0.00008800
O	-2.46789000	-0.05040300	-1.13342300
O	-2.46732600	-0.04838500	1.13363500
O	0.12354400	1.76167500	-0.00021600
C	1.08642000	2.78624400	-0.00034400
H	0.53053900	3.72145100	-0.00045100
H	1.72415100	2.74992100	0.89003000
H	1.72413800	2.74970200	-0.89072100

Anion 2b

Sum of electronic and thermal Free energies = -534.868580 a.u.
E (RB3LYP) = -534.968511685 a.u.
Sum of electronic and thermal Enthalpies = -534.822220 a.u.

C	0.87195100	1.49404200	-0.00001900
C	-0.40957400	2.02198100	0.00001200
C	-1.53197200	1.18657800	0.00001700
C	-1.33749500	-0.19330600	-0.00000900
C	-0.04822600	-0.72410400	-0.00003900
C	1.06499500	0.10748200	-0.00004500
H	1.75130400	2.12207900	-0.00002600
H	-0.55686800	3.09598500	0.00003200
H	-2.52166900	1.61866100	0.00004200
H	0.10790800	-1.79363600	-0.00005800
C	2.49650700	-0.49558600	-0.00008400
O	2.55336700	-1.74633000	0.00005600
O	3.42786000	0.34233400	0.00005200
O	-2.37131800	-1.11491600	-0.00000600
C	-3.69163000	-0.62304000	0.00002000
H	-4.34189800	-1.49568900	0.00001500
H	-3.90268600	-0.02020400	0.88958100
H	-3.90271100	-0.02017500	-0.88951500

Anion 2c

Sum of electronic and thermal Free energies = -534.871058 a.u.
E (RB3LYP) = -534.969171878 a.u.
Sum of electronic and thermal Enthalpies = -534.824836 a.u.

C	0.57717900	1.27058900	0.00001200
C	-0.80313900	1.41708500	0.00000300
C	-1.62210100	0.28733800	-0.00000800
C	-1.04772400	-0.98181100	-0.00000900
C	0.34405800	-1.10395200	0.00000200
C	1.17819100	0.00845500	0.00001200
H	1.23116800	2.13202600	0.00001900
H	-1.26726100	2.39518200	0.00000400
H	-1.65998500	-1.87223800	-0.00001800
H	0.81414400	-2.07803400	0.00000300
C	2.71838200	-0.14492700	0.00002600
O	3.14280300	-1.32457600	-0.00000800
O	3.36436900	0.92934100	-0.00001900
O	-2.98602000	0.52771500	-0.00001900
C	-3.84735900	-0.58954200	0.00001100
H	-3.70574400	-1.21175600	0.88921500
H	-4.86068900	-0.19263700	0.00002000
H	-3.70577300	-1.21178500	-0.88917700

Anion 3a

Sum of electronic and thermal Free energies = -475.710976 a.u.
E (RB3LYP) = -475.796308280 a.u.
Sum of electronic and thermal Enthalpies = -475.668973 a.u.

C	0.23826800	-0.26465900	-0.02854200
C	-0.51458600	-1.43698800	0.00260000
C	-1.90649900	-1.43417400	0.03643600

C	-2.57678500	-0.21206800	0.03794200
C	-1.85822900	0.97354200	-0.00634900
C	-0.45176200	0.97351500	-0.04519400
H	0.04594200	-2.36193700	0.00923000
H	-2.45870900	-2.36517000	0.06550600
H	-3.65993000	-0.18035600	0.06797500
H	-2.38037500	1.92440400	-0.02484700
C	1.77961300	-0.37481800	0.00784500
O	2.42045500	0.70936000	0.16080500
O	2.25544300	-1.52168600	-0.09663500
N	0.24664200	2.16840200	-0.14608900
H	-0.18459600	2.95544500	0.31079100
H	1.24387100	2.00131000	0.05218300

Anion 3b

Sum of electronic and thermal Free energies = -475.701596 a.u.
E (RB3LYP) = -475.785945371 a.u.
Sum of electronic and thermal Enthalpies = -475.658400 a.u.

C	-0.62839200	0.12674000	-0.00865500
C	-0.29045500	1.48225800	0.00542800
C	1.04522400	1.86824600	0.00871400
C	2.06303400	0.91568400	-0.00529300
C	1.73651500	-0.44216300	-0.01761200
C	0.39316400	-0.81917200	-0.02085300
H	-1.09580200	2.20263600	0.01655100
H	1.30687100	2.92035300	0.02125600
H	3.10318900	1.22250900	-0.01393400
H	0.10067500	-1.86238500	-0.02750400
C	-2.11209000	-0.32997300	0.00233700
O	-2.29258100	-1.57054600	0.01225800
O	-2.95853100	0.59311600	0.00234600
N	2.76111300	-1.42595800	-0.08147000
H	3.58747600	-1.16375200	0.43640000
H	2.43669600	-2.32794000	0.23629100

Anion 3c

Sum of electronic and thermal Free energies = -475.700607 a.u.
E (RB3LYP) = -475.784932367 a.u.
Sum of electronic and thermal Enthalpies = -475.657388 a.u.

C	-0.74670700	-0.00004100	-0.00644200
C	-0.02410900	1.19265500	-0.01042600
C	1.36774300	1.20047500	-0.01477700
C	2.08120300	0.00000200	-0.01341000
C	1.36775400	-1.20046000	-0.01483000
C	-0.02410000	-1.19267300	-0.01045900
H	-0.58846100	2.11534900	-0.00674700
H	1.90865200	2.14159600	-0.02102900
H	1.90870300	-2.14156500	-0.02115100
H	-0.58848100	-2.11537400	-0.00680100
C	-2.29374600	-0.00002400	0.00709300
O	-2.83314500	-1.13198700	0.01249500
O	-2.83299800	1.13205200	0.01240600
N	3.50209500	-0.00002600	-0.06840500
H	3.90296100	0.82340500	0.35729400

H	3.90287500	-0.82335700	0.35755700

Anion 4a

Sum of electronic and thermal Free energies = -757.491786 a.u.
E (RB3LYP) = -757.56221838 a.u.
Sum of electronic and thermal Enthalpies = -757.445872 a.u.

C	-0.92259600	0.34063500	0.00000600
C	-2.22487900	-0.17016900	0.00000700
C	-2.49742700	-1.53005100	0.00000900
C	-1.44532300	-2.43801700	0.00000500
C	-0.14113800	-1.96404200	0.00000100
C	0.13615500	-0.59082700	0.00000300
H	-3.01160000	0.57080700	0.00000600
H	-3.52289100	-1.88011500	0.00001100
H	-1.63014700	-3.50472800	0.00000500
H	0.67454500	-2.66937000	-0.00000400
C	-0.79842100	1.90746000	0.00000800
O	0.35662300	2.36634800	0.00004400
O	-1.89773400	2.50649700	-0.00004500
C	1.60622800	-0.20487100	-0.00000400
F	1.99705900	0.46916000	1.09312700
F	1.99702800	0.46923000	-1.09310200
F	2.39962200	-1.33839700	-0.00005000

Anion 4b

Sum of electronic and thermal Free energies = -757.507749 a.u.
E (RB3LYP) = -757.57729204 a.u.
Sum of electronic and thermal Enthalpies = -757.460822 a.u.

C	-1.56451500	0.14157300	-0.00013600
C	-1.60907400	1.53339700	-0.00013700
C	-0.43806300	2.28994600	-0.00017300
C	0.79918600	1.66109100	-0.00019900
C	0.85273200	0.26436900	-0.00022100
C	-0.31844300	-0.48752700	-0.00018400
H	-2.58642700	1.99624300	-0.00011200
H	-0.48806300	3.37188600	-0.00018700
H	1.71244500	2.24093900	-0.00023700
H	-0.30660000	-1.56737200	-0.00019300
C	-2.87134600	-0.69697700	-0.00006900
O	-2.70598000	-1.93691300	0.00021900
O	-3.92929500	-0.02884500	0.00026500
C	2.19785900	-0.38832900	0.00000500
F	2.95068400	-0.03518600	1.08346100
F	2.14801800	-1.73495900	-0.00188400
F	2.95250200	-0.03217600	-1.08118300

Anion 4c

Sum of electronic and thermal Free energies = -757.511382 a.u.
E (RB3LYP) = -757.57814467 a.u.
Sum of electronic and thermal Enthalpies = -757.460686 a.u.

C	-1.78620500	0.00172700	-0.00489900
C	-1.07644100	1.20244200	-0.01221300

C	0.31334900	1.21613800	-0.02514500
C	1.01758300	0.01207700	-0.03491900
C	0.32088600	-1.19842000	-0.02619600
C	-1.06727200	-1.19535600	-0.01264200
H	-1.64992800	2.11884100	-0.00919200
H	0.85135000	2.15386100	-0.03407800
H	0.86574300	-2.13320300	-0.03653200
H	-1.63479200	-2.11544500	-0.00980300
C	-3.33865500	-0.00402700	0.00849800
O	-3.86460700	-1.13940000	0.01358100
O	-3.87323500	1.12722300	0.01293100
C	2.50902300	0.00319100	-0.00052300
F	3.01208600	-0.21946000	1.25023200
F	3.05173300	-0.96958300	-0.78018400
F	3.06026600	1.17201200	-0.41163300

Anion 5a

Sum of electronic and thermal Free energies = -624.912727 a.u.
E (RB3LYP) = -624.978913573 a.u.
Sum of electronic and thermal Enthalpies = -624.864800 a.u.

C	0.04492200	-0.73265800	0.00065900
C	-0.93484800	-1.73545300	0.02887100
C	-2.28951800	-1.44300000	0.02996400
C	-2.72431800	-0.11644000	-0.01056600
C	-1.78440600	0.89973800	-0.04643000
C	-0.42728500	0.58072500	-0.04759700
H	-0.59475700	-2.76170400	0.04439800
H	-3.01387200	-2.24785700	0.05863100
H	-3.77975300	0.12013600	-0.02150000
H	-2.07842000	1.93776100	-0.08268700
C	1.52150900	-1.17168600	0.13877900
O	1.91546600	-1.94214600	-0.75772800
O	2.07920700	-0.75421500	1.17100200
N	0.50269600	1.71826700	-0.13557400
O	0.15954500	2.77601800	0.40418700
O	1.53473100	1.57489800	-0.76894800

Anion 5b

Sum of electronic and thermal Free energies = -624.926774 a.u.
E (RB3LYP) = -624.994053664 a.u.
Sum of electronic and thermal Enthalpies = -624.879700 a.u.

C	-1.22781300	0.14186300	-0.00001400
C	-1.19026500	1.53594600	-0.00001400
C	0.02091000	2.22815600	0.00000800
C	1.22230100	1.53548100	0.00002500
C	1.17684700	0.14184000	0.00002200
C	-0.02436400	-0.55972100	0.00000500
H	-2.14101500	2.05128400	-0.00003600
H	0.03023900	3.31079600	0.00001200
H	2.17466100	2.04059400	0.00004200
H	-0.05418800	-1.63788500	0.00000900
C	-2.58553800	-0.61351500	-0.00003700
O	-2.49731500	-1.85944100	0.00007000

O	-3.59579000	0.12447900	-0.00003200
N	2.44567600	-0.59581400	0.00003700
O	2.41087700	-1.82289500	-0.00005900
O	3.49699100	0.05105900	-0.00001100

Anion 5c

Sum of electronic and thermal Free energies = -624.928978 a.u.
E (RB3LYP) = -624.996264165 a.u.
Sum of electronic and thermal Enthalpies = -624.881797 a.u.

C	-1.41610900	0.00010900	0.00001100
C	-0.70326600	1.20301400	-0.00000800
C	0.68263900	1.21595900	-0.00001800
C	1.36561900	0.00000800	-0.00000100
C	0.68259100	-1.21595900	0.00002000
C	-0.70326200	-1.20288500	0.00002300
H	-1.27669100	2.11904600	-0.00001200
H	1.24241900	2.13820300	-0.00003400
H	1.24235400	-2.13821300	0.00003200
H	-1.27671600	-2.11892100	0.00003400
C	-2.96878900	-0.00009200	0.00002100
O	-3.49584100	-1.13400900	-0.00007100
O	-3.49579800	1.13396300	0.00004300
N	2.82401200	-0.00001800	-0.00000700
O	3.41232200	-1.08359600	0.00001500
O	3.41231800	1.08352800	-0.00001900

Anion 6a

Sum of electronic and thermal Free energies = -1306.149160 a.u.
E (RB3LYP) = -1306.22362698 a.u.
Sum of electronic and thermal Enthalpies = -1306.092783 a.u.

C	-1.60675600	0.52274300	0.02706000
C	-2.94925000	0.32828000	-0.30353400
C	-3.47092300	-0.93841500	-0.52647700
C	-2.65418900	-2.05900200	-0.40174500
C	-1.32117900	-1.89882800	-0.04837900
C	-0.80845800	-0.61700200	0.16186000
H	-3.55656600	1.21941800	-0.37816100
H	-4.51310000	-1.05495800	-0.79749500
H	-3.04843200	-3.05239300	-0.57054900
H	-0.67583700	-2.75317800	0.07871400
C	-1.07786300	1.97039300	0.14105600
O	0.15481700	2.06731200	-0.03635300
O	-1.93842900	2.84552800	0.35067900
S	0.90419500	-0.59731400	0.76733500
O	1.07658600	0.19876000	1.96007500
O	1.31591700	-1.99812600	0.80996300
C	2.04633400	0.10068400	-0.62321500
F	2.65361800	1.21442600	-0.26104700
F	1.39534900	0.25971400	-1.77361400
F	3.00763300	-0.82555900	-0.83695600

Anion 6b

Sum of electronic and thermal Free energies = -1306.164266 a.u.

E (RB3LYP) = -1306.23684066 a.u.
Sum of electronic and thermal Enthalpies = -1306.105936 a.u

C	-2.37872800	0.09249100	0.00900100
C	-2.57854100	1.41717900	0.39610000
C	-1.53553200	2.34270400	0.39623800
C	-0.25983600	1.95580700	0.01245700
C	-0.06457000	0.62759000	-0.37464700
C	-1.10205100	-0.30410300	-0.38296200
H	-3.58174800	1.69149400	0.69283100
H	-1.71499300	3.36808900	0.69328000
H	0.56348300	2.65342500	-0.00563800
H	-0.95804900	-1.32898500	-0.69240600
C	-3.55622000	-0.92285800	0.01509200
O	-3.25532000	-2.07773100	-0.35123300
O	-4.65136600	-0.44944600	0.38863300
S	1.56058000	0.13236800	-0.85468700
O	1.52681900	-1.02013100	-1.72971400
O	2.37558000	1.29351000	-1.16488700
C	2.31106100	-0.52738800	0.75418900
F	1.62197000	-1.56966800	1.21990200
F	2.33507100	0.42496400	1.69604600
F	3.56993900	-0.91752400	0.51646500

Anion 6c

Sum of electronic and thermal Free energies = -1306.166269 a.u.
E (RB3LYP) = -1306.23880257 a.u.
Sum of electronic and thermal Enthalpies = -1306.107801 a.u.

C	2.68064700	0.00013400	-0.02291400
C	1.98447200	1.20483000	-0.15462900
C	0.62213000	1.22011800	-0.41132000
C	-0.05008900	0.00173800	-0.54106000
C	0.62190200	-1.21750700	-0.41756500
C	1.98415800	-1.20380500	-0.16073500
H	2.55158200	2.11910700	-0.05137500
H	0.08129100	2.14811500	-0.52536400
H	0.08084600	-2.14477100	-0.53643200
H	2.55101700	-2.11873800	-0.06207300
C	4.20722700	-0.00091200	0.26823600
O	4.72241400	-1.13546700	0.36451000
O	4.72239500	1.13304600	0.36975600
S	-1.77800300	0.00269000	-0.85363200
O	-2.20453100	1.26950900	-1.41625600
O	-2.20452600	-1.26061100	-1.42415900
C	-2.54550400	-0.00273100	0.87719300
F	-2.17881900	-1.09074200	1.56281600
F	-2.17862100	1.08079600	1.56973000
F	-3.87948800	-0.00225100	0.76293300

Computed Hirshfeld atomic charges

Substituted benzoic acids with N electrons

Atoms	1a	1b	1c	2a	2b	2c	3a	3b	3c
C_1	0.2158	0.2153	0.2136	0.2097	0.2171	0.2101	0.2083	0.2162	0.2069
C_2	-0.0189	-0.0167	-0.0206	-0.0285	-0.0143	-0.0299	-0.0406	-0.0169	-0.0352
C_3	0.0240	-0.0329	-0.0268	0.0939	-0.0404	-0.0242	0.0725	-0.0505	-0.0259
C_4	-0.0465	0.0078	-0.0455	-0.0685	0.0777	-0.0655	-0.0667	0.0533	-0.0636
C_5	-0.0315	-0.0359	0.0168	-0.0311	-0.0576	0.0869	-0.0322	-0.0540	0.0630
C_6	-0.0471	-0.0422	-0.0471	-0.0584	-0.0414	-0.0562	-0.0676	-0.0425	-0.0655
C_7	-0.0341	-0.0375	-0.0327	-0.0316	-0.0494	-0.0304	-0.0349	-0.0535	-0.0319
C_8	-0.0985	-0.0929	-0.0922	-0.0037	-0.0076	-0.0056			
O_1	-0.2792	-0.2936	-0.2954	-0.2918	-0.2913	-0.3006	-0.2873	-0.2953	-0.3045
O_2	-0.1832	-0.1824	-0.1838	-0.1893	-0.1814	-0.1859	-0.1819	-0.1816	-0.1810
O_3				-0.1174	-0.1367	-0.1320			
N_1							-0.1705	-0.1927	-0.1810
H_1	0.1822	0.1840	0.1835	0.1793	0.1844	0.1824	0.1823	0.1836	0.1804
H_2	0.0462	0.0489	0.0488	0.0451	0.0566	0.0507	0.0435	0.0470	0.0492
H_3	0.0514	0.0489	0.0468	0.0516	0.0467	0.0537	0.0506	0.0458	0.0444
H_4	0.0495	0.0493	0.0480	0.0477	0.0501	0.0468	0.0444	0.0494	0.0453
H_5	0.0462	0.0482	0.0528	0.0476	0.0464	0.0537	0.0465	0.0449	0.0532
H_6	0.0436	0.0436	0.0442	0.0576	0.0548	0.0453	0.1285	0.1221	0.1272
H_7	0.0436	0.0442	0.0432	0.0438	0.0420	0.0553	0.1051	0.1244	0.1274
H_8	0.0365	0.0436	0.0463	0.0438	0.0430	0.0453			

Atoms	4a	4b	4c	5a	5b	5c	6a	6b	6c
C_1	0.2169	0.2188	0.2195	0.2196	0.2203	0.2214	0.2196	0.2210	0.2224
C_2	-0.0060	-0.0088	-0.0057	0.0028	-0.0059	0.0005	0.0107	-0.0037	0.0040
C_3	-0.0092	-0.0204	-0.0246	0.0468	-0.0117	-0.0159	-0.0276	-0.0080	-0.0135
C_4	-0.0354	-0.0226	-0.0334	-0.0250	0.0417	-0.0238	-0.0204	-0.0290	-0.0193
C_5	-0.0232	-0.0217	-0.0158	-0.0237	-0.0161	0.0478	-0.0250	-0.0117	-0.0231
C_6	-0.0295	-0.0317	-0.0305	-0.0217	-0.0284	-0.0250	-0.0153	-0.0257	-0.0205
C_7	-0.0265	-0.0207	-0.0186	-0.0198	-0.0143	-0.0220	-0.0248	-0.0105	-0.0196
C_8	0.2294	0.2291	0.2295				0.2102	0.2088	0.2090
O_1	-0.2706	-0.2836	-0.2840	-0.2784	-0.2787	-0.2795	-0.2717	-0.2766	-0.2774
O_2	-0.1782	-0.1779	-0.1769	-0.1781	-0.1762	-0.1742	-0.1733	-0.1752	-0.1729
O_3				-0.1956	-0.1911	-0.1933	-0.2884	-0.2918	-0.2946
O_4				-0.1903	-0.1974	-0.1942	-0.2952	-0.2962	-0.2941
N_1				0.2257	0.2262	0.2268			
S_1							0.4569	0.4575	0.4580
F_1	-0.0903	-0.0980	-0.0983				-0.0599	-0.0690	-0.0703
F_2	-0.0903	-0.0813	-0.0922				-0.0733	-0.0723	-0.0708
F_3	-0.0919	-0.0980	-0.0850				-0.0605	-0.0587	-0.0585
H_1	0.1875	0.1888	0.1891	0.1939	0.1908	0.1913	0.1942	0.1918	0.1924
H_2	0.0512	0.0566	0.0548	0.0611	0.0620	0.0575	0.0611	0.0648	0.0586
H_3	0.0581	0.0592	0.0551	0.0605	0.0610	0.0602	0.0609	0.0643	0.0635
H_4	0.0571	0.0572	0.0582	0.0603	0.0602	0.0609	0.0615	0.0616	0.0642
H_5	0.0509	0.0548	0.0588	0.0616	0.0575	0.0614	0.0605	0.0587	0.0625

Substituted benzoic acids with N+1 electrons

Atoms	1a	1b	1c	2a	2b	2c	3a	3b	3c
C_1	0.1143	0.1155	0.1171	0.1154	0.1120	0.1416	0.1056	0.1128	0.1572
C_2	-0.0810	-0.0812	-0.0819	-0.0912	-0.0802	-0.0682	-0.0890	-0.0804	-0.0677
C_3	-0.0287	-0.0911	-0.0991	0.0486	-0.1196	-0.0740	0.0292	-0.1225	-0.0704
C_4	-0.1014	-0.0384	-0.1002	-0.1290	0.0343	-0.0971	-0.1356	0.0092	-0.1021
C_5	-0.1422	-0.1409	-0.0676	-0.1431	-0.1523	0.0367	-0.1407	-0.1517	0.0166
C_6	-0.1059	-0.1020	-0.0991	-0.1128	-0.1061	-0.0920	-0.1192	-0.1063	-0.0982
C_7	-0.1036	-0.1053	-0.0890	-0.1103	-0.1021	-0.0713	-0.1145	-0.1093	-0.0634
C_8	-0.1196	-0.1167	-0.1262	-0.0242	-0.0266	-0.0899			
O_1	-0.3932	-0.4095	-0.4077	-0.4053	-0.4101	-0.3789	-0.3985	-0.4116	-0.3665
O_2	-0.2377	-0.2368	-0.2362	-0.2389	-0.2396	-0.2246	-0.2362	-0.2387	-0.2178
O_3				-0.1366	-0.1600	-0.1608			
N_1							-0.2105	-0.2250	-0.2066
H_1	0.1365	0.1389	0.1397	0.1354	0.1379	0.1502	0.1373	0.1373	0.1528
H_2	0.0086	0.0136	0.0087	0.0088	0.0143	0.0236	0.0002	0.0053	0.0219
H_3	-0.0088	-0.0096	0.0067	-0.0085	-0.0020	0.0239	-0.0099	-0.0106	-0.0178
H_4	0.0077	0.0081	0.0073	0.0074	0.0068	0.0125	0.0042	0.0060	-0.0096
H_5	0.0056	0.0076	0.0137	0.0042	0.0105	0.0225	0.0025	0.0078	0.0274
H_6	0.0199	0.0125	0.0043	0.0345	0.0309	-0.0446	0.0936	0.0888	-0.0819
H_7	0.0199	0.0224	0.0095	0.0229	0.0259	-0.0909	0.0814	0.0883	-0.0742
H_8	0.0094	0.0125	-0.0001	0.0229	0.0259	-0.0187			

Atoms	4a	4b	4c	5a	5b	5c	6a	6b	6c
C_1	0.1256	0.1245	0.1272	0.1949	0.1860	0.1667	0.2035	0.1530	0.1485
C_2	-0.0779	-0.0787	-0.0774	-0.0453	-0.0509	-0.0640	-0.0521	-0.0659	-0.0678
C_3	-0.0750	-0.0708	-0.0933	0.0085	-0.0411	-0.0712	-0.0970	-0.0374	-0.0774
C_4	-0.0834	-0.0750	-0.0940	-0.0693	0.0032	-0.0766	-0.0819	-0.0790	-0.0791
C_5	-0.1300	-0.1334	-0.1030	-0.0835	-0.0917	0.0028	-0.0937	-0.1179	-0.0903
C_6	-0.1023	-0.0911	-0.0915	-0.1050	-0.0769	-0.0746	-0.1265	-0.0778	-0.0772
C_7	-0.0851	-0.1012	-0.0814	-0.0605	-0.1069	-0.0646	-0.0735	-0.1098	-0.0708
C_8	0.2073	0.2099	0.1995				0.1827	0.1906	0.1874
O_1	-0.3784	-0.3985	-0.3962	-0.3400	-0.3364	-0.3576	-0.3192	-0.3696	-0.3732
O_2	-0.2300	-0.2296	-0.2289	-0.1961	-0.2014	-0.2094	-0.1960	-0.2140	-0.2171
O_3				-0.3548	-0.3395	-0.3333	-0.3403	-0.3350	-0.3423
O_4				-0.3403	-0.3411	-0.3342	-0.3509	-0.3369	-0.3418
N_1				0.1321	0.1388	0.1454			
S_1							0.3834	0.4127	0.4001
F_1	-0.1182	-0.1245	-0.1356				-0.0784	-0.0825	-0.0859
F_2	-0.1182	-0.1052	-0.1256				-0.0932	-0.0864	-0.0864
F_3	-0.1224	-0.1245	-0.1144				-0.1009	-0.0906	-0.0934
H_1	0.1438	0.1446	0.1453	0.1622	0.1621	0.1584	0.1613	0.1548	0.1541
H_2	0.0177	0.0248	0.0145	0.0311	0.0390	0.0255	0.0262	0.0418	0.0240
H_3	0.0008	0.0029	0.0160	0.0211	0.0205	0.0279	0.0177	0.0158	0.0298
H_4	0.0111	0.0157	0.0185	0.0137	0.0244	0.0289	0.0029	0.0240	0.0309
H_5	0.0146	0.0102	0.0202	0.0308	0.0118	0.0298	0.0260	0.0096	0.0281

Substituted benzoic acids with N-1 electrons

Atoms	1a	1b	1c	2a	2b	2c	3a	3b	3c
C_1	0.2332	0.2314	0.2420	0.2285	0.2305	0.2373	0.2331	0.2304	0.2351
C_2	0.0386	0.0427	0.1003	0.0404	0.0294	0.0776	0.0248	0.0303	0.0710
C_3	0.1342	0.0413	0.0356	0.1654	0.0419	0.0332	0.1264	0.0255	0.0224
C_4	0.0395	0.1285	0.0216	0.0063	0.1578	-0.0002	0.0157	0.1249	0.0102
C_5	0.0292	0.0344	0.1285	0.0283	0.0054	0.1616	0.0185	0.0158	0.1284
C_6	0.0970	0.0292	0.0340	0.0692	0.0298	0.0295	0.0552	0.0180	0.0128
C_7	0.0386	0.0990	0.0245	0.0217	0.0705	0.0161	0.0243	0.0631	0.0172
C_8	-0.0587	-0.0521	-0.0536	0.0305	0.0285	0.0284			
O_1	-0.2259	-0.2371	-0.2038	-0.2375	-0.2431	-0.2195	-0.2363	-0.2469	-0.2248
O_2	-0.1575	-0.1577	-0.1518	-0.1616	-0.1596	-0.1555	-0.1486	-0.1588	-0.1585
O_3				0.0009	-0.0085	-0.0137			
N_1							-0.0134	-0.0267	-0.0285
H_1	0.2108	0.2135	0.2151	0.2079	0.2114	0.2123	0.2123	0.2109	0.2106
H_2	0.0900	0.0872	0.0861	0.0817	0.0942	0.0847	0.0838	0.0824	0.0834
H_3	0.0940	0.0905	0.0898	0.0917	0.0822	0.0956	0.0892	0.0845	0.0843
H_4	0.1054	0.0940	0.0885	0.0999	0.0925	0.0822	0.0965	0.0901	0.0842
H_5	0.0856	0.0981	0.0898	0.0823	0.0933	0.0885	0.0824	0.0915	0.0867
H_6	0.0895	0.0912	0.0813	0.0896	0.0882	0.0774	0.1849	0.1819	0.1825
H_7	0.0895	0.0747	0.0762	0.0773	0.0777	0.0874	0.1513	0.1828	0.1829
H_8	0.0670	0.0912	0.0960	0.0773	0.0777	0.0774			

Atoms	4a	4b	4c	5a	5b	5c	6a	6b	6c
C_1	0.2315	0.2492	0.2521	0.2566	0.2543	0.2409	0.2630	0.2568	0.2401
C_2	0.0320	0.1084	0.1063	0.0771	0.0755	0.0503	0.0842	0.0787	0.0476
C_3	0.1074	0.0687	0.0701	0.0977	0.0793	0.1207	0.0006	0.0716	0.1188
C_4	0.0768	0.0197	0.0159	0.0175	0.0715	0.0570	0.0521	-0.0024	0.0670
C_5	0.0264	0.1110	0.0955	0.0787	0.0781	0.0923	0.0885	0.0835	0.0142
C_6	0.1076	0.0736	0.0604	0.0512	0.0827	0.1051	0.0308	0.0727	0.1049
C_7	0.0831	0.0271	0.0251	0.0212	0.0263	0.0526	0.0474	0.0273	0.0590
C_8	0.2468	0.2407	0.2471				0.2197	0.2249	0.2216
O_1	-0.2169	-0.1799	-0.1724	-0.1306	-0.1643	-0.2093	-0.1037	-0.1572	-0.2126
O_2	-0.1515	-0.1434	-0.1391	-0.1294	-0.1336	-0.1444	-0.1119	-0.1309	-0.1445
O_3				-0.0979	-0.1134	-0.1234	-0.2613	-0.2495	-0.2387
O_4				-0.1037	-0.1163	-0.1148	-0.2558	-0.2365	-0.2504
N_1				0.2457	0.2438	0.2417			
S_1							0.4707	0.4822	0.4801
F_1	-0.0516	-0.0704	-0.0570				-0.0548	-0.0535	-0.0563
F_2	-0.0516	-0.0566	-0.0561				-0.0597	-0.0547	-0.0566
F_3	-0.0598	-0.0704	-0.0529				-0.0346	-0.0283	-0.0297
H_1	0.2166	0.2208	0.2226	0.2271	0.2227	0.2200	0.2297	0.2236	0.2201
H_2	0.0969	0.0943	0.0969	0.0926	0.0955	0.0966	0.0954	0.0954	0.0972
H_3	0.0993	0.1097	0.0917	0.1047	0.1004	0.1058	0.1070	0.1016	0.1069
H_4	0.1117	0.1063	0.1011	0.1000	0.1075	0.1002	0.0973	0.1051	0.1030
H_5	0.0950	0.0910	0.0926	0.0913	0.0899	0.1086	0.0953	0.0895	0.1082

Electronegativity and Global Softness

Electronegativity (eV)

Benzoic acids	1	2	3	4	5	6
**a**	4.5337	4.1803	3.8855	5.1136	5.5469	5.4444
**b**	4.5534	4.2241	3.9573	5.1959	5.6893	5.5630
**c**	4.5168	4.1790	3.8947	5.2949	5.8478	5.7976

Global softness (eV^{-1})

Benzoic acids	1	2	3	4	5	6
**a**	0.1149	0.1227	0.1307	0.1158	0.1239	0.1213
**b**	0.1151	0.1240	0.1313	0.1120	0.1195	0.1158
**c**	0.1144	0.1206	0.1261	0.1142	0.1254	0.1208

Condensed Fukui function calculated using Hirshfeld charge

Electrophilic attack

Atoms	1a	1b	1c	2a	2b	2c	3a	3b	3c
C_1	0.017	0.016	0.028	0.019	0.013	0.027	0.025	0.014	0.028
C_2	0.058	0.059	0.121	0.069	0.044	0.110	0.065	0.047	0.110
C_3	0.110	0.074	0.062	0.071	0.082	0.057	0.054	0.076	0.049
C_4	0.086	0.121	0.067	0.075	0.080	0.065	0.082	0.072	0.074
C_5	0.061	0.070	0.112	0.059	0.063	0.075	0.051	0.070	0.066
C_6	0.144	0.071	0.081	0.130	0.071	0.086	0.123	0.061	0.079
C_7	0.073	0.136	0.057	0.053	0.120	0.046	0.059	0.120	0.050
C_8	0.040	0.041	0.039	0.034	0.036	0.034			
O_1	0.053	0.056	0.092	0.054	0.048	0.081	0.051	0.048	0.080
O_2	0.026	0.025	0.032	0.028	0.022	0.030	0.033	0.023	0.023
O_3				0.118	0.128	0.118			
N_1							0.157	0.166	0.154
H_1	0.029	0.029	0.032	0.029	0.027	0.030	0.030	0.027	0.030
H_2	0.044	0.038	0.037	0.037	0.038	0.034	0.040	0.035	0.035
H_3	0.043	0.042	0.043	0.040	0.036	0.042	0.039	0.039	0.040
H_4	0.056	0.045	0.040	0.052	0.042	0.035	0.052	0.041	0.039
H_5	0.039	0.050	0.037	0.035	0.047	0.035	0.036	0.047	0.034
H_6	0.046	0.048	0.037	0.032	0.033	0.032	0.056	0.060	0.056
H_7	0.046	0.031	0.033	0.033	0.033	0.032	0.046	0.058	0.056
H_8	0.030	0.048	0.050	0.033	0.035	0.032			

Atoms	4a	4b	4c	5a	5b	5c	6a	6b	6c
C_1	0.091	0.094	0.092	0.037	0.034	0.020	0.043	0.036	0.018
C_2	0.072	0.070	0.072	0.074	0.081	0.050	0.073	0.082	0.044
C_3	0.066	0.050	0.069	0.051	0.091	0.137	0.028	0.080	0.132
C_4	0.048	0.052	0.061	0.043	0.030	0.081	0.073	0.027	0.086
C_5	0.107	0.112	0.087	0.102	0.094	0.044	0.114	0.095	0.037
C_6	0.073	0.059	0.061	0.073	0.111	0.130	0.046	0.098	0.125
C_7	0.059	0.080	0.063	0.041	0.041	0.074	0.072	0.038	0.079
C_8	0.022	0.019	0.030				0.010	0.016	0.013
O_1	0.108	0.115	0.112	0.148	0.114	0.070	0.168	0.119	0.065
O_2	0.052	0.052	0.052	0.049	0.043	0.030	0.061	0.044	0.028
O_3				0.098	0.078	0.070	0.027	0.042	0.056
O_4				0.087	0.081	0.079	0.039	0.060	0.044
N_1				0.020	0.018	0.015			
S_1							0.014	0.025	0.022
F_1	0.028	0.027	0.037				0.018	0.013	0.016
F_2	0.028	0.024	0.033				0.020	0.014	0.016
F_3	0.030	0.027	0.029				0.040	0.032	0.035
H_1	0.029	0.032	0.033	0.033	0.032	0.029	0.035	0.032	0.028
H_2	0.046	0.038	0.042	0.031	0.034	0.039	0.034	0.031	0.039
H_3	0.041	0.050	0.037	0.044	0.039	0.046	0.046	0.037	0.043
H_4	0.055	0.049	0.043	0.040	0.047	0.039	0.036	0.044	0.039
H_5	0.044	0.036	0.034	0.030	0.032	0.047	0.035	0.031	0.046

Nucleophilic attack

Atoms	1a	1b	1c	2a	2b	2c	3a	3b	3c
C_1	0.102	0.100	0.097	0.094	0.105	0.069	0.103	0.103	0.049
C_2	0.062	0.065	0.061	0.063	0.066	0.038	0.048	0.063	0.032
C_3	0.053	0.058	0.072	0.045	0.079	0.050	0.043	0.072	0.044
C_4	0.055	0.046	0.055	0.061	0.043	0.032	0.069	0.044	0.038
C_5	0.111	0.105	0.084	0.112	0.095	0.050	0.108	0.098	0.046
C_6	0.059	0.060	0.052	0.054	0.065	0.036	0.052	0.064	0.032
C_7	0.069	0.068	0.056	0.079	0.053	0.041	0.080	0.056	0.031
C_8	0.021	0.024	0.034	0.021	0.019	0.084			
O_1	0.114	0.116	0.112	0.113	0.119	0.078	0.111	0.116	0.062
O_2	0.054	0.054	0.052	0.050	0.058	0.039	0.054	0.057	0.036
O_3				0.019	0.023	0.029			
N_1							0.040	0.032	0.025
H_1	0.046	0.045	0.044	0.044	0.047	0.032	0.045	0.046	0.027
H_2	0.038	0.035	0.039	0.036	0.042	0.027	0.043	0.042	0.027
H_3	0.060	0.059	0.041	0.060	0.049	0.030	0.061	0.056	0.062
H_4	0.042	0.041	0.040	0.040	0.043	0.034	0.040	0.043	0.054
H_5	0.041	0.041	0.040	0.043	0.036	0.031	0.044	0.037	0.026
H_6	0.024	0.031	0.040	0.023	0.024	0.090	0.035	0.033	0.207
H_7	0.024	0.022	0.034	0.021	0.016	0.146	0.024	0.036	0.200
H_8	0.027	0.031	0.046	0.021	0.017	0.064			

Atoms	4a	4b	4c	5a	5b	5c	6a	6b	6c
C_1	0.091	0.094	0.092	0.025	0.034	0.055	0.016	0.068	0.074
C_2	0.072	0.070	0.072	0.048	0.045	0.064	0.063	0.062	0.072
C_3	0.066	0.050	0.069	0.038	0.029	0.055	0.069	0.029	0.064
C_4	0.048	0.052	0.061	0.044	0.038	0.053	0.061	0.050	0.060
C_5	0.107	0.112	0.087	0.060	0.076	0.045	0.069	0.106	0.067
C_6	0.073	0.059	0.061	0.083	0.049	0.050	0.111	0.052	0.057
C_7	0.059	0.080	0.063	0.041	0.093	0.043	0.049	0.099	0.051
C_8	0.022	0.019	0.030				0.028	0.018	0.022
O_1	0.108	0.115	0.112	0.062	0.058	0.078	0.047	0.093	0.096
O_2	0.052	0.052	0.052	0.018	0.025	0.035	0.023	0.039	0.044
O_3				0.159	0.148	0.140	0.052	0.043	0.048
O_4				0.150	0.144	0.140	0.056	0.041	0.048
N_1				0.094	0.087	0.081			
S_1							0.074	0.045	0.058
F_1	0.028	0.027	0.037				0.018	0.013	0.016
F_2	0.028	0.024	0.033				0.020	0.014	0.016
F_3	0.030	0.027	0.029				0.040	0.032	0.035
H_1	0.044	0.044	0.044	0.032	0.029	0.033	0.033	0.037	0.038
H_2	0.033	0.032	0.040	0.030	0.023	0.032	0.035	0.023	0.035
H_3	0.057	0.056	0.039	0.039	0.041	0.032	0.043	0.049	0.034
H_4	0.046	0.042	0.040	0.047	0.036	0.032	0.059	0.038	0.033
H_5	0.036	0.045	0.039	0.031	0.046	0.032	0.035	0.049	0.034

Absolute gas phase acidities for **1a–6c** calculated at the M062X/6-311++G(2d,2p) level of theory. The values are reported in kcal/mol. $\Delta_{acid}G° = G°(anion) + G°(H^+) − G°(acid)$. The experimental values are given in parentheses and were obtained from http://webbook.nist.gov/chemistry/

	**1**	**2**	**3**	**4**	**5**	**6**
a	338.58 (332.46 ± 2.01)	342.80 (332.46 ± 2.01)	337.03 (330.31 ± 2.01)	332.39 (-------)	329.73 (324.33 ± 2.01)	322.5 (-----)
b	339.47 (333.65 ± 2.01)	339.43 (332.46 ± 2.01)	340.47 (334.61 ± 2.01)	331.50 (325.29 ± 2.01)	327.77 (322.18 ± 2.01)	324.86 (-----)
c	339.96 (333.89 ± 2.01)	340.74 (333.89 ± 2.01)	342.81 (336.28 ± 2.01)	330.78 (325.29 ± 2.01)	326.63 (320.98 ± 2.01)	323.50 (-----)

Absolute gas phase acidities for **1a–6c** calculated at the MP2/6-311++G(2d,2p) level of theory. The values are reported in kcal/mol. $\Delta_{acid}G° = G°(anion) + G°(H^+) − G°(acid)$. The experimental values are given in parentheses and were obtained from http://webbook.nist.gov/chemistry/

	**1**	**2**	**3**	**4**	**5**	**6**
a	331.64 (332.46 ± 2.01)	332.50 (332.46 ± 2.01)	328.23 (330.31 ± 2.01)	324.61 (-------)	321.63 (324.33 ± 2.01)	320.09 (-----)
b	331.70 (333.65 ± 2.01)	332.46 (332.46 ± 2.01)	333.98 (334.61 ± 2.01)	323.31 (325.29 ± 2.01)	319.79 (322.18 ± 2.01)	317.42 (-----)
c	332.95 (333.89 ± 2.01)	332.26 (333.89 ± 2.01)	333.88 (336.28 ± 2.01)	323.29 (325.29 ± 2.01)	319.77 (320.98 ± 2.01)	316.82 (-----)
